# Validating the Digital Health Literacy Instrument in Relation to COVID-19 Information (COVID-DHL-K) among South Korean Undergraduates

**DOI:** 10.3390/ijerph19063437

**Published:** 2022-03-14

**Authors:** Heeran Chun, Eun-Ja Park, Seul Ki Choi, Hyeran Yoon, Orkan Okan, Kevin Dadaczynski

**Affiliations:** 1 Department of Health Administration, Jungwon University, Chungbuk 28024, Korea; yhr0809@naver.com; 2 Korea Institute for Health Social Affairs, Sejong 30147, Korea; ejpark@kihasa.re.kr; 3 Graduate School of Urban Public Health, University of Seoul, Seoul 02504, Korea; skchoi@uos.ac.kr; 4 Department of Sports and Health Sciences, Technical University Munich, 80809 Munich, Germany; orkan.okan@tum.de; 5 Department of Nursing and Health Science, Fulda University of Applied Sciences, 36037 Fulda, Germany; kevin.dadaczynski@pg.hs-fulda.de; 6 Center for Applied Health Sciences, Leuphana University Lueneburg, 21335 Lueneburg, Germany

**Keywords:** digital health literacy, measure, validation, university students

## Abstract

Digital health literacy is crucial in accessing and applying health information in the COVID-19 pandemic period. Young college students are exposed daily to digital technologies, and they have further increased the use of digital information during the COVID-19 period. This study aimed to adapt DHLI into Korean and to assess the psychometric properties, during the COVID-19 pandemic period. A cross-sectional, nationwide, and web-based survey was conducted among 604 Korean undergraduates from 23 December 2020 to 8 January 2021. On the basis of the Digital Health Literacy Instrument (DHLI) by the Global COVID HL Network, the Korean questionnaire was developed by group translation, expert reviews, and forward–backward translation for validation. The scale reliability and validity were examined using Cronbach’s alpha and confirmatory factor analysis. Results support the theoretical and empirical four-factor structure (search, express, evaluate, use) in the coronavirus-related DHL among Korean University students. Internal reliability of the overall scale was high (Cronbach’s α = 0.908). The four-factor model was supported by confirmatory factor analysis (GFI = 0.972, CFI = 0.984, TLI = 0.978, RMSEA = 0.045). This study revealed that the COVID-DHL-K is a valid and reliable measure with appropriate psychometric characteristics.

## 1. Introduction

The coronavirus disease 2019 (COVID-19) has been challenging, and health literacy is an important resource in the battle to overcome the worldwide coronavirus disease pandemic [1,2]. As most measures and guidance for COVID-19 have been released online, digital health literacy is crucial in accessing and applying health information in the COVID pandemic period. Much information is accessible through the Internet, but it does not guarantee the appropriate use of information. The “infodemic”, information epidemic, makes it more difficult for people to pick up the correct health information [3,4]. Social media is rife with infodemic “rumors”, “stigma”, and “conspiracy theories” related to the coronavirus. A recent study revealed that the “infodemic” is so prevalent that 82% of the text ratings analyzed (1856 of the 2276 reports) were classified as being “false” [3]. 

It is noteworthy that the level of digital health literacy has associations with health status [5,6], as well as on one’s trust in and satisfaction with the information received and evaluated [7]. Digital health literacy can be a problem among young adults, because young people, such as university students, are easily regarded as being proficient with digital technology, called “digital natives”. However, some young university students may experience difficulties in finding, understanding, and utilizing information [8]. There exists inequity in digital literacy, and the “digital divide” was exacerbated in the pandemic period, as shown by urban–rural differences or socioeconomic inequalities [9,10,11]. Previous studies found a digital health literacy disparity and its association with attitudes and preventive behaviors toward COVID-19 [8,12,13].

To measure digital health literacy, eHEALS [14] was used globally. As eHEALS focused on “information searching” as the first generation of web use, the Digital Health Literacy Instrument (DHLI) [15] was introduced. The new tool DHLI can include interactivities on the web, so-called “e-Health 2.0 Skills” [15]. The Korean version of the eHealth Literacy Scale (K-eHEALS) was validated among young adults [16] and among older adults [17,18]. Among older people, the validated K-DHLI, composed of 21 items with five factors, was recently introduced [18]. Previous studies reported an association between digital health literacy and doctor–patient communication [19], as well as preventive health behaviors [20,21]. However, internationally comparable validated measures for digital health literacy among young adults during the COVID-19 period have rarely been studied despite the pro-digital environment of Korea. Almost all young adults (100% in the 20s and 99% in the 30s) use smartphones in South Korea [22], and this has further increased the digital information during the pandemic period. The Korean version of the Digital Health Literacy Instrument among university students would be valuable in the development of related policy aiming to increase digital health literacy and compliance with the policies meant to control COVID-19. 

In this context, the objective of this study was to develop a Korean version of the Digital Health Literacy Instrument (DHLI) in relation to the COVID-19 period in a sample of Korean undergraduate students. 

## 2. Materials and Methods

### 2.1. Study Design and Samples

A nationwide web-based survey using a snowball sample design was conducted among Korean university students, using a Google Forms survey. All the data were electronically recorded. The survey link was restricted to one email account having only one participation attempt, in order to prevent duplicate answers. For homogeneity of participants’ demographic or environmental characteristics, the survey included undergraduate students only. As inclusion criteria, we limited this survey to “undergraduate students”, who were studying in domestically located universities and colleges. Hence, students who were attending university at that time or taking a leave of absence were included. The number of university students in South Korea is about 2.5 million, occupying a large portion of the young population. They are savvy in interacting with social media, being the early adopters of digital devices such as smartphones and iPads. They are also familiar with digital contents, because many offline lectures were replaced with online ones during the COVID-19 pandemic in South Korea. These were the reasons why the survey was administered online. The duration of the survey was about 2 weeks from 23 December 2020 to 8 January 2021. During the period of the survey, cumulative numbers of coronavirus confirmed cases increased from 52,548 to 67,359. This period was during the third-wave outbreak of the coronavirus pandemic in South Korea. The study was approved by the JW University ethics committee (1044297-HR-202010-012-02). Participants for this survey were recruited through the encouragement and direction of acquaintances and colleagues who contributed to the research team. The survey links were shared with students at 20 universities nationwide, which were first posted on the department webpage or the school’s intranet of students’ associations. Participation was voluntary, and the survey was started only after confirmation of the consent form. Participants were informed about their rights to confidentiality, privacy, and withdrawal from the study at any time. 

### 2.2. Measures/Questionnaire

The Global COVID-HL Network adapted a coronavirus-related DHLI of a total of 15 items, modifying the originally developed version by van der Vaart and Drossaert [15]. The original DHLI was composed of seven subscales (a total of 21 items). While reviewing the DHLI measurement, the network team agreed that two subscales (operational and navigational skills) did not fit well with young adults. A “ceiling effect” was expected for these two items and for this target group. In turn, in the original study [15] surveyed a much older sample with almost 50% of respondents aged 50 and older. Operational skills include the ability to use the keyboard, the mouse, links etc., and navigational skills include the frequency of losing track of a website or not knowing how to return to a previous page. These all are fundamental skills for media use, and as young people currently grow up with these skills, the network did not see a benefit in including these two subscales. The coronavirus-related DHLI used includes five subscales:

(1) Searching the web for information on coronavirus (e.g., making a choice; using a proper word or search query; finding exact information),
(2) Adding self-generated content on coronavirus (e.g., clearly formulating questions or concerns; expressing opinion or feelings; writing a message to make people understand exactly),
(3) Evaluating the reliability of coronavirus-related information (e.g., deciding whether the information is reliable or not; deciding whether it is with commercial interests or not; checking different websites for the same information),
(4) Determining personal relevance of coronavirus-related information (e.g., deciding if the information is applicable; applying the information in daily life; using the information about health),
(5) Protecting privacy on the Internet (e.g., finding it difficult to judge who can read along; sharing one’s own private information; sharing someone else’s private information).

On the basis of the DHLI by the network, the Korean questionnaire was developed by group translation, expert reviews, and forward–backward translation for validation. The primary translation was conducted by three researchers who majored in public health. Next, the research team met to review the clarity of the translated tool, as well as the appropriateness of expressions and vocabulary, and revised it where necessary. Later, the reverse translation was requested by a researcher who had never seen the questionnaire and was fluent in both English and Korean to maintain mutual independence between translation and reverse translation. Next, a reverse translator and three researchers met to check whether there were any differences in the meaning of the items in the translated tools, and whether there were problems such as distortion due to ambiguity in expression or cultural differences.

In the process of adaptation to the Korean language and culture, the scales were entitled (1) search, (2) express, (3) evaluate, (4) use, and (5) protect privacy. After a series of statistical analyses and discussions, we chose a four-factor model. The fifth factor “protect privacy” was not included in the final Korean DHL measure, as further explained in Section 3. Each scale consisted of three items and could be answered on a four-point response scale from very difficult to very easy. Mean values were calculated for each DHLI subscale.

Sociodemographic information included gender, age, location of the university, number of trusted confidants to talk to in need of a difficult situation (more than three, two, one, or none), subjective social status (low from 1 to 4, middle from 5 to 7, and high from 8 to 10, using the MacArthur scale by Adler et al. [23]), and sufficient pocket money (completely sufficient, sufficient, not sufficient). We used the sufficiency of pocket money as a proxy indicator of household income. College students may not know the exact household income; thus, we used the variable as an indicator of economic status. Other coronavirus-related information-seeking behaviors and health problems were also assessed. As health-related variables for this study, we used the WHO-5 index, which consists of five questions including a six-point Likert scale from 0–5, with higher values indicating a higher wellbeing. The WHO-5 wellbeing items include having felt “cheerful, active, or refreshed when waking up”. The total sum score of this measure can be used as continuous variable or it can be dichotomized for screening depression (a cutoff score of ≤50) [24,25].

### 2.3. Data Analysis

A descriptive analysis was conducted to show the characteristics of study participants and the mean differences in DHL score according to the group. Each of the 12 items were in the ±1 range of skewedness and kurtosis, and no floor or ceiling effect could be detected. Bivariate analyses were conducted for comparing means in the total scores of the DHL according to the subgroups. Next, Pearson and Spearman coefficient correlation was used to test the internal reliability of the scales and the hypothesized associations with sociodemographic and health condition variables. To assess the validity of the four-factor DHL in Korean, confirmatory factor analysis was conducted, using the maximum-likelihood estimation. Construct validity was tested by confirmatory factor analysis, theoretical (known groups) validity, and convergent–discriminant validity. Measures composed of subscales are recommended to conduct confirmatory factor analysis for the factor structure [26].

## 3. Results

### 3.1. Descriptive Analysis

The sample consisted of 604 undergraduate students, studying in South Korea (Table 1). Of the participants, 72.2% were women and 27.8% were men. Most of the participants were in the 20–24-year-old category (72.2%). While 68.5% of the respondents reported having more than three trusted confidants to talk to, 31% reported only having two, one, or none. About 68% of them had sufficient pocket money, but 32% reported their pocket money as being insufficient. Surprisingly, 61.6% of students indicated symptoms of depression according to the WHO wellbeing index (a cutoff score of ≤50).

Table 1 shows that the mean DHL score of 12 items was 2.91 (±0.49). A higher DHL score was observed for younger respondents (*p* = 0.014). A higher level of social support appeared to be associated with an increase in DHL score (*p* < 0.001). Higher subjective social status and sufficient pocket money showed significant associations with high overall DHL score (*p* < 0.05). Students with (very) low wellbeing had a lower DHL score, compared to those with sufficient wellbeing. Gender and residence were not systematically associated with DHL.

### 3.2. Reliability and Validity

This research chose a four-factor model, in line with theoretical and statistical associations. Considering the negative and positive associations with other subscales, the fifth subscale, privacy protection, was not included in the final measure in the Korean study. In this study, the reliability of this domain was acceptable (Cronbach’s α = 0.818), but the theoretical validity was not supported, showing a negative association with two domains (*r* = −0.033 for “express” and *r* = −0.052 for “evaluate”) and a positive association with two domains (*r* = 0.031 for “search” and *r* = 0.052 for “use”). In addition, items of privacy protection did not fully support the normality assumption (kurtosis = −1.151 for item 13 and skewedness = 1.086 for item 15), with a ceiling effect (53%, 55%, and 64% for the highest category in the items 13, 14, and 15; see Supplementary Table S1).

The internal reliability of overall DHL in the Korean language was high (Cronbach’s α = 0.908), and construct validity was satisfied. For the reliability of subdimensions, Cronbach’s alpha coefficients ranged from “express, α = 0.861”, to “search, α = 0.844”, “use, α = 0.838”, and “evaluate, α = 0.768”.

**Figure 1:**
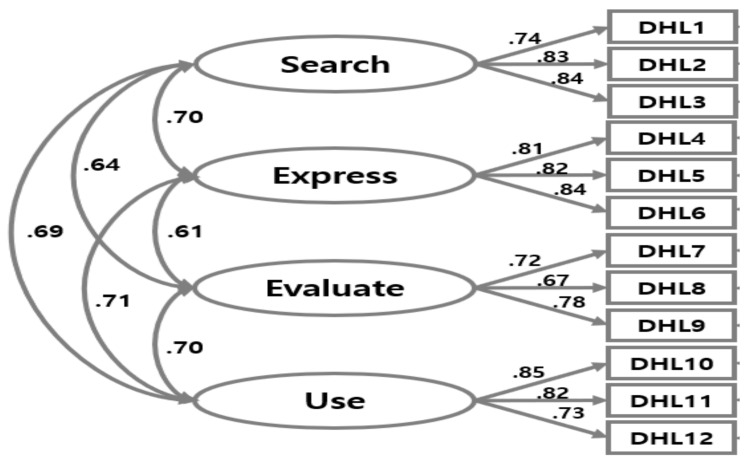
Results of confirmatory factor analysis for the COVID-DHL-K.

Figure 1 and Table 2 and Table 3 depict the results of the confirmatory factor analysis to establish the construct validity. Items were loaded onto four theoretical and hypothetical factors related to “search” (factor 1), “express” (factor 2), “evaluate” (factor 3), and “use” (factor 4) in coronavirus-related digital health information. Convergent validity was satisfactory; the standardized regression coefficients of each item were all in an acceptable range (Β = 0.67–0.85), supporting that each item was well constructed for the latent factor. Confirmatory analysis for the four-factor model (Table 2) showed excellent goodness-of-fit indices (χ^2^ = 107.383, df = 48, GFI = 0.972, IFI = 0.984, CFI = 0.984, TLI = 0.978, RMSEA = 0.045 (0.034–0.057), SRMR = 0.027) [27,28]. While the five-factor model had an acceptable model fit, it was not supported by theoretical associations, where both negative and positive associations between privacy protection factor and other factors were present.

Additional statistics for construct validity were satisfactory. The AVE values for the four latent factors ranged from 0.809 to 0.680 (AVE ≥ 0.5), and the CR values of the four factors ranged from 0.926 to 0.864 (CR ≥ 0.7) (Table 3). Discriminant validity was also satisfactory as the AVE values of all four latent factors were greater than the square of the surrounding latent factor correlation coefficient (Table 3).

## 4. Discussion

The study showed that the COVID-DHL-K is a valid and reliable measure for capturing the DHL proficiency of South Korean university students. The measure supported the four psychometric factor structures without floor/ceiling effects, validated by a high level of internal reliability and construct validity with satisfactory model fit indices. This measure can be used for international comparisons in the level and patterns of DHL with regard to health seeking behaviors of university students.

The Korean validated comprehensive digital health literacy (COVID-DHL-K) measure, originally developed in the Netherlands [15], highlights the need for a broad range of skills for health-related internet use. The study reveals that the new tool can include interactivities on the web, compared with the existing DHL tool which only focuses on searching for digital health information. This DHL measure also incorporated the validation method by evaluating the self-reported digital use on the basis of the actual utilization ability of people. The Global COVID-HL research network slightly modified this tool to measure the web-based health information available during the pandemic. Previous studies in the United States [12] and in South Korea [21] revealed the relationship between the types of digital health information searched for by college students and its influence on their preventive health behaviors during COVID-19. In the context of sharing and expressing opinions on rapidly changing coronavirus-related information on social media, a comprehensive DHL tool is crucial.

The Korean DHL measure did not include the subscale on privacy protection from the Global COVID-HL core questionnaire [8]. In the descriptive analysis for the DHL subscales, all three items in the “privacy protection” category showed high ceiling effects (53%, 55%, and 64%). This means that more than half of the survey participants did not have any difficulty in posting information online using social network sites (SNS) regarding “privacy protection”. Moreover, previous studies [8,29] reported low reliability for this subscale. A recent Portuguese study [29] also supported “the four-factor model for DHL” belonging to the COVID-HL network and used the same adapted version of the DHLI.

The Korean COVID-DHL measure was tested by the confirmatory factor analysis for validity. Construct validity, by convergent and discriminant validity, and theoretical validity were satisfactory. Convergent validity was satisfactory by AVE and CR (average variance extracted and critical ratio) (AVE ≥ 0.5; CR ≥ 0.7). The criterion validity was satisfactory, as the total sum of the Korean DHL was negatively associated with age and positively associated with higher social status, higher social support, and higher mental health status as measured by the WHO wellbeing index. The correlation between a higher DHL and frequency of mental health problems is also in line with previous studies [6,30], implying that e-Health 2.0 skills have a positive effect on actual health status. In another study, DHL played a mediation role between individual factors and health behaviors [31].

This study had some limitations. Firstly, it is difficult to apply the results to Korean adults as convenience sampling of university students was conducted. Secondly, due to the nature of online surveys not having personal contact information, we could not proceed with test–retest reliability. Thirdly, these associations between DHL and health should be interpreted with caution due to the inherent traits of cross-sectional design. However, this study had a proper sample size of 604 students collected from across the country. The strength of this study is its investigation of a comprehensive DHL measure, including various digital information sources and types of information searched by college students with regard to COVID-19. Moreover, this study systematically tested the reliability and validity of the tool by performing confirmatory factor analysis. A recent study [5] emphasized the importance of a measurement-based approach for digital health equity.

## 5. Conclusions

The COVID-DHL-K proved to be a feasible and valid tool to assess young adults’ DHL. The DHL tool we used was designed for international comparisons, and the results in the Korean survey are consistent with the studies of the adapted DHLI from Global COVID-HL Network. As a result, we suggest that the 12-item DHL tool consisting of three questions each with four factor structures ((1) search, (2) express, (3) evaluate, (4) use) was statistically supported. There should be further studies to measure the levels and the patterns of DHL among Koreans, with representative samples, as well as to explore social disparities in DHL in the population groups. We believe that COVID-DHL-K can serve its purpose as a validated measure for digital health literacy. It was developed in the context of COVID-19 in relation to university students, but it can be easily modified for other health conditions and can be adapted for other age groups.

## Figures and Tables

**Table 1:** General characteristics and group comparisons in the mean of total digital health literacy.

				DHLI Score			
Variables		*n*	%	M	±SD	t/F	*p*-Value
Total				2.91	0.49		
Gender	Men	146	24.2	2.93	0.60	0.384	0.701
	Women	458	75.8	2.91	0.44		
Age	<20	104	17.2	3.02	0.48	4.331	0.014
	20–24	436	72.2	2.90	0.48		
	25+	64	10.6	2.81	0.51		
Residence	Metropolitan	216	35.8	2.91	0.48	0.003	0.997
	City	313	51.8	2.91	0.49		
	Rural (eup, myon)	75	12.4	2.91	0.53		
Trusted confidant	None, One	85	14.1	2.76	0.45	7.336	0.001
	Two	105	17.4	2.84	0.47		
	More than three	414	68.5	2.96	0.49		
Subjective social status	High (8–10)	73	12.1	3.07	0.51	4.224	0.015
	Middle (5–7)	379	62.7	2.89	0.50		
	Low (1–4)	152	25.2	2.89	0.44		
Pocket money	Completely sufficient	54	8.9	3.13	0.57	6.512	0.020
	Sufficient	356	58.9	2.86	0.46		
	Not sufficient	194	32.1	2.94	0.50		
WHO wellbeing	Sufficient	232	38.4	3.02	0.49	4.425	<0.001
	Very low (≤50)	372	61.6	2.84	0.48		

**Table 2:** Confirmatory factor analysis models fit indices (*n* = 604).

Model	χ^2^	df	*p*	GFI	CFI	TLI	RMSEA	SRMR
Five-factor model	174.060	80	<0.001	0.964	0.979	0.973	0.044 (0.035–0.053)	0.031
Four-factor model	107.383	48	<0.001	0.972	0.984	0.978	0.045 (0.034–0.057)	0.027

**Table 3:** Results of convergent and discriminant validity for the COVID-DHL-K.

	Search	Express	Evaluate	Use	AVE	CR
Search	0.817				0.817	0.930
Express	0.699 (0.489)	0.809			0.809	0.927
Evaluate	0.640 (0.410)	0.608 (0.370)	0.664		0.664	0.855
Use	0.692 (0.479)	0.713 (0.508)	0.703 (0.494)	0.806	0.806	0.926

## Supplementary Materials

The following are available online at https://www.mdpi.com/article/10.3390/ijerph19063437/s1: Table S1. Distribution of DHL-K measure.
