# Supplementary material for: Validating the Digital Health Literacy Instrument in Relation to COVID-19 Information (COVID-DHL-K) among South Korean Undergraduates
Source: Int J Environ Res Public Health. 2022 Mar 14;19(6):3437. doi: 10.3390/ijerph19063437 (PMC8950100; doi:10.3390/ijerph19063437)
Supplement: Supplementary file 1 [file ijerph-19-03437-s001.zip › ijerph-1603187-supplementary.pdf]

Table S1. Distribution of COVID-DHL-K measure (n = 604)

	Domain	Distribution (%)				Mean	SD	Min	Max	Skewness	Kurtosis	Cronbach's Alpha	
		Very Difficult	Fairly Difficult	Fairly Easy	Very Easy							α = 0.908 * α = 0.840 **	Deleted by
DHL1	Search	1.5	17.2	65.2	16.1	2.96	0.63	1	4	−0.338	0.642	0.844	0.821
DHL2		1.0	9.6	62.6	26.8	3.15	0.62	1	4	−0.362	0.611		0.752
DHL3		1.8	19.4	56.6	22.2	2.99	0.70	1	4	−0.310	−0.041		0.773
DHL4	Express	1.7	19.0	61.3	18.0	2.96	0.66	1	4	−0.301	0.264	0.861	0.817
DHL5		2.8	21.4	55.6	20.2	2.93	0.72	1	4	−0.344	−0.013		0.800
DHL6		2.5	25.7	53.3	18.5	2.88	0.73	1	4	−0.202	−0.269		0.798
DHL7	Evaluate	6.6	40.6	42.9	9.9	2.56	0.76	1	4	0.019	−0.368	0.768	0.699
DHL8		4.3	32.9	49.5	13.2	2.72	0.74	1	4	−0.117	−0.310		0.694
DHL9		3.6	34.4	49.0	12.9	2.71	0.73	1	4	−0.049	−0.349		0.672
DHL10	Use	1.5	18.2	59.3	21.0	3.00	0.67	1	4	−0.293	0.096	0.838	0.745
DHL11		1.7	15.2	62.1	21.0	3.02	0.65	1	4	−0.381	0.485		0.745
DHL12		1.2	13.9	61.6	23.3	3.07	0.64	1	4	−0.328	0.342		0.831
DHL13	Protect privacy	6.6	28.1	11.9	53.3	3.12	1.03	1	4	−0.063	−1.151	0.818	0.828
DHL14		5.6	23.2	15.9	55.3	3.21	0.98	1	4	0.784	−0.800		0.684
DHL15		5.1	19.2	11.8	63.9	3.34	0.96	1	4	−1.086	−0.265		0.733

*: Cronbach's α value of DHL1 to 12; **: Cronbach's α valueof DHL1 to 15.
